# How Collective Efficacy Mediates the Association between Principal Instructional Leadership and Teacher Self-Efficacy: Findings from a Meta-Analytic Structural Equation Modeling (MASEM) Study

**DOI:** 10.3390/bs14020085

**Published:** 2024-01-25

**Authors:** Turgut Karakose, Abdurrahman Kardas, Sedat Kanadlı, Tijen Tülübaş, Bilal Yildirim

**Affiliations:** 1 Faculty of Education, Kutahya Dumlupınar University, 43100 Kütahya, Türkiye; tijen.tulubas@dpu.edu.tr; 2 Batman Provincial Directorate of National Education, 72070 Batman, Türkiye; abkardas@gmail.com; 3 Faculty of Education, Mersin University, 33100 Mersin, Türkiye; skanadli@mersin.edu.tr; 4 Necatibey Faculty of Education, Balikesir University, 10145 Balikesir, Türkiye; bildirim61@gmail.com

**Keywords:** principal instructional leadership, instructional leadership, collective efficacy, teacher self-efficacy, leadership, schools, MASEM

## Abstract

Principal instructional leadership (PIL) is significant for school effectiveness due to its direct and indirect influences on school-, teacher-, and student-level variables. A considerable number of studies have provided persuasive evidence that PIL is associated with both collective efficacy (CEF) and teacher self-efficacy (TSEF), two significant variables to sustain the quality of instruction. These studies were conducted with a variety of participants from various contexts. The current study aims to investigate the association between PIL and TSEF, and the mediating role of CEF in this association using meta-analytical structural equation modeling (MASEM). This analysis was conducted using the correlation values obtained from 26 studies focusing on their relationship and included data from a population of 19.584 participants from around the world, thus providing a more generalizable perspective on these variables. The results indicated that PIL was correlated with both CEF and TSEF, and the combined influence of PIL and CEF on TSEF was 31%. The study findings also showed that the scales used to measure PIL could produce different results regarding these relationships, while gender did not have a significant effect. These results suggest significant implications for researchers, policymakers, and practitioners to sustain school effectiveness in the fast-changing context of schools in the twenty-first century.

## 1. Introduction

The sustainability of schools in the twenty-first century requires effective leadership and teaching practices. In their rapidly changing environment, the goals and tasks of schools are rapidly changing. Hence, the competencies and efficacy of educational professionals have become even more important in training students as citizens of a future society [1,2,3]. Research has continuously shown that school leadership is vital in increasing the instructional capacity of schools for sustained improvement and effective learner outcomes [4,5,6]. In their seminal study, Leithwood et al. [7] stated that school leadership is second only to teaching in improving student outcomes, and most leadership effects occur indirectly via their direct effects on people and processes at school [8,9,10,11].

Starting from the very early years of effective school research, studies have provided evidence indicating that principals who devote most of their time and efforts to improving instruction rather than managerial matters are highly capable of promoting instructional quality and student achievement [12,13,14,15]. This line of research particularly focused on the significance of instructional leadership, which is demarcated from other leadership models with its closer interest in teaching and learning activities [16]. It showed that principals engaging in instructional leadership practices such as supervising teachers and students, managing the curriculum, or identifying and resolving instructional problems could significantly influence teachers’ beliefs, perceptions, and quality of instruction [17,18,19]. ‘By articulating an inspiring vision of learning for the school, setting challenging but attainable goals, clarifying standards of teacher and pupil performance, fostering teacher learning and development, and coaching teachers for success’ [20] (p. 6), these principals are found to promote the collective efficacy and self-efficacy of teachers, two significant determiners of teacher and student achievement [5,18,21,22]. Therefore, these two variables—collective teacher efficacy (CEF) and teacher self-efficacy (TSEF)—have become points of interest as the components of the emotional path associating school leadership with student achievement [9,10,23].

TSEF refers to a teacher’s confidence in organizing and practicing necessary actions to attain educational goals, and it impacts a teacher’s emotions, actions, attitudes, cognitive strategies, coping behaviors, and choice of actions [24,25]. Thus, TSEF determines a teacher’s efforts to achieve goals, their ability to face and cope with the challenges of teaching, and maintain their higher expectations from even the unmotivated students [17,26]. However, considering that teachers often do not work alone but share the responsibility to attain educational goals, their belief in teachers’ collective efficacy to provide these courses of action also matters. In such a school environment, the collaboration of principals and teachers as well as the collective involvement of teachers in the development of curricula and learning materials improve the collective expertise of schools and promote better student outcomes [27,28]. Evidence exists in the literature indicating that PIL is significantly related to both TSEF and CEF [22,29,30], which in turn improves the quality of instruction and promotes student outcomes [11,31].

Since research has validated the indirect effects of school leadership on student outcomes, particularly the stronger relationship between PIL and student achievement in comparison to other leadership models [11], understanding the pathway from PIL to student achievement has become a significant point of research to understand the mechanisms that impact school success and effectiveness. Although previous research has shed some light on these factors, a more global investigation would help produce more generalizable results. The present study aims to fulfill this purpose and focuses on the associations between PIL, TSEF, and CEF as three significant determiners of school success. In particular, this study aims to test a model that represents the role of CEF in the association between PIL and TSE and evaluates the association between (a) PIL and CEF, (b) PIL and TSEF, and (c) TSEF and CEF, as well as (d) the mediating role of CEF in the association between PIL and TSEF using meta-analytic structural equation modeling (MASEM) in which the weighted effect sizes between these variables are measured using the results of previous research. Thus, the study significantly contributes to the literature by providing a more global understanding of their association.

## 2. Conceptual Background

### 2.1. Instructional Leadership

The theory of instructional leadership is empirically situated in the field of research on school effectiveness, with studies initially undertaken in Western contexts and later conducted more globally [32,33]. As a model of leadership that emerged from the educational literature, it has become a target of extensive scholarly interest, particularly due to its close relationship with improvement in teaching and learning [34,35,36].

The earlier conceptualization of PIL by Hallinger and Murphy [37] has become prevalent in the literature, and over 500 studies were conducted using this framework and its associated instrument—the Principal Instructional Management Rating Scale (PIMRS) [38]. The framework includes three main dimensions with a total of ten subdimensions. The first of these dimensions refers to the school’s mission and goals, while the other two refer to managing the instructional program and developing a positive learning climate [34,37]. Regarding the first dimension, principals are expected to communicate the goals and vision of the school to the whole school community and set a clear direction to accomplish them. The second dimension, on the other hand, encompasses the principal’s efforts to develop and coordinate the curriculum, supervise and evaluate teacher instruction, and monitor student progress. The third dimension is related to developing a positive school climate and culture that fosters student learning, which basically includes protecting instructional time, providing material and non-material assistance for improving teaching and learning, nourishing the collective efforts of teachers to improve instruction, and providing opportunities for continuous professional development. By engaging in these leadership actions, the principal acts as ‘an inspector, director, and supervisor of educational issues’ [39] (p. 2) and aims to improve the learning of all students [35] by promoting teachers’ beliefs and actions to accomplish this goal.

### 2.2. Teacher Self-Efficacy

Recognizing the role of beliefs and cognition in teacher agency, educational scholars have developed the concept of TSEF based on Bandura’s seminal definition of self-efficacy as ‘beliefs in one’s capabilities to organize and execute courses of action required to manage prospective situations’ [40] (p. 2). Similarly, TSEF is defined as ‘individual teachers’ beliefs in their own abilities to plan, organize, and carry out activities required to attain given educational goals’ [41] (p. 612). Like self-efficacy, TSEF is shaped by environmental conditions and impacts teachers’ attitudes and behaviors toward educational goals [42]. TSEF encompasses both activity and outcome expectancy; the former refers to a teacher’s beliefs in their ability to plan and engage in necessary actions to achieve goals, and the latter refers to the extent to which these beliefs reinforce the teacher’s efforts and performance to fulfill targeted tasks [26,43]. As a result, TSEF significantly influences the capacity of a school to achieve better teaching and learning outcomes [17,44].

According to Bandura’s conceptualization of self-efficacy [26], TSEF develops over four major sources: enactive mastery experiences (e.g., positive experiences with teaching/learning), vicarious experiences (e. g., observations of others’ experiences), verbal persuasion (e.g., verbal information by others about their efficacy as teachers), and emotional and physiological reactions (e.g., stress, well-being, commitment, and motivation). Among these, enactive mastery experiences are considered to be ‘the most influential source of self-efficacy because they provide the most authentic evidence of whether one can master whatever it takes to succeed’ [26] (p. 80). Accordingly, TSEF can be closely linked to teachers’ experiences with instruction, classroom management, how to motivate students, and how to communicate with other teachers or parents [45,46].

TSEF was initially conceptualized as a one-dimensional construct indicating teachers’ beliefs in their overall teaching ability but was later developed into a three-dimensional construct that included instruction-specific components by Tschannen-Moran and Hoy [47]. In their widely used instrument called the Teachers’ Sense of Efficacy Scale (TSES), Tschannen-Moran and Hoy [47] measured TSEF in enabling student engagement, practicing effective classroom management, and using instructional strategies properly, which has been empirically tested and validated several times [10].

### 2.3. Collective Efficacy

While self-efficacy is related to an individual’s belief in his/her ability to engage in necessary actions to accomplish a goal, CEF refers to ‘the shared group belief related to the abilities of organizing and managing the action steps necessary for acquiring skills at different levels’ [26] (p. 477). CEF stems from the perceptions of teachers about their shared ability to achieve desired student outcomes [48] and is significant in reinforcing the strength and capacity of teachers altogether [25]. CEF is considered a ‘promising construct for promoting understanding of ways schools can foster student achievement’ [48] (p. 189) and comprises teachers’ beliefs in the group’s ability not only to engage in goal-targeted performance but also to cope with the challenges they face in this process [28]. Research has shown that perceived CEF is a significant variable in ensuring school improvement and effectiveness by promoting student achievement [27,48,49,50].

### 2.4. The Proposed Model of the Study

The educational leadership literature has provided significant findings pointing to a close association between principal leadership and teacher behaviors [28,51]. Principal instructional leadership is considered to provide significant foundations for the development of TSEF and CEF by promoting their competence in enacting their teaching roles effectively and setting structures to support schoolwide instruction [30,52].

Both TSEF and CEF were built on the basis of social cognitive theory, which fundamentally suggests that human behavior is shaped through the reciprocal relationships between cognition, attitudes, and environmental factors [26]. On this basis, TSEF and CEF are mainly shaped by how teachers make sense of their experiences, as well as other teachers’ experiences, and their reactions to environmental conditions [28]. Research suggests that PIL may be one of these significant environmental conditions as it influences the lived experiences of teachers individually and as a whole by monitoring, supporting, and supervising instructional processes, as well as by contributing to teacher professional development. As a result, PIL can change teachers’ perceptions about their ability to achieve educational goals both at the individual and collective levels [30,53,54,55].

There is also a significant body of evidence indicating that principals engaging in instructional leadership can promote CEF ‘by articulating an inspiring vision of learning for the school, setting attainable goals with teachers, clarifying standards of teacher and pupil performance, clarifying how teacher actions can positively impact students learning, and coaching teachers for success’ [20] (p. 6). PIL also supports CEF by building a culture of cooperation and collaboration, which creates a schoolwide synergy to facilitate instructional improvement and student achievement [46,48,56,57]. As suggested by Goddard et al. [28], PIL can provide ‘a form of social persuasion that can positively influence collective efficacy beliefs’, and ‘enhance the resolve of teachers that they possess the ability necessary to achieve student learning goals’ (p. 504).

Several studies have also investigated the association between CEF and TSEF and provided persuasive evidence of their positive correlation [58,59,60]. As suggested by Skaalvik and Skaalvik [41], CEF could support TSEF by providing an encouraging and persistent culture of being achievement-oriented even in the face of complex challenges. As shown by other studies, social engagement and supportive relationships among teachers positively affect individual teaching experiences and promote professional growth, which is likely to stimulate TSEF [27,61,62] and help them persist in their efforts to achieve goals [58].

**Figure 1:**
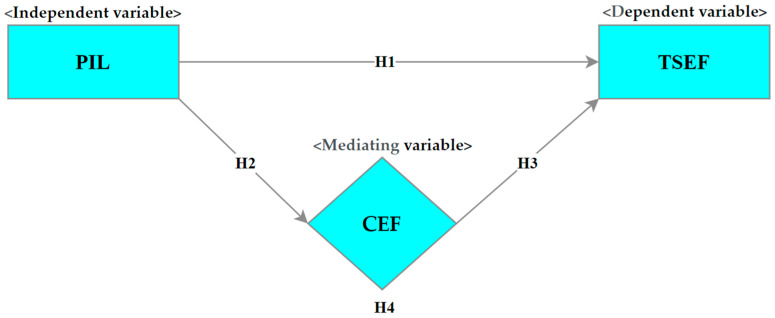
Proposed theoretical model. (Note. PIL = principal instructional leadership; TSEF = teacher self-efficacy; CEF = collective efficacy).

The literature on school effectiveness suggests that all three variables, namely PIL, CEF, and TSEF, significantly contribute to school effectiveness, and their interaction can promote the process of establishing effective schools [22,57,63]. On this basis, the current study suggests four specific hypotheses that are tested using a meta-analytic structural equation modeling (MASEM) approach: (***H1***) There is a significant positive association between PIL and TSEF; (***H2***) there is a significant positive association between PIL and CEF; (***H3***) there is a positive and significant association between TSEF and CEF; and (***H4***) CEF moderates the association between PIL and TSEF. The proposed model of the study is presented in Figure 1.

## 3. Materials and Methods

### 3.1. Research Design and Data Collection

In the current study, we used meta-analytic structural equation modeling (MASEM) to assess the relationships among PIL, CEF, and TSEF. MASEM is a novel and advanced method of testing the casual relationships among the variables in a proposed model of research by calculating correlations and multiple regressions [64,65,66]. As we aimed to assess the association between PIL and TSEF, and the mediating role of CEF in this association through the analysis of existing empirical results in the literature, MASEM was a suitable methodology to employ.

Data collection for review and meta-analytic studies is often conducted using electronic databases and following the PRISMA protocol, which includes the stages of identification, screening, eligibility, and inclusion of data sources [67]. Data search for the current study was conducted on 4 September 2023 using three major databases, namely Web of Science (WoS), Scopus, and Google Scholar, without making any time specifications. We searched these databases for novel research papers published in journals following rigorous scientific publishing procedures such as being cited or indexed in locally or internationally renowned databases. This was significant to support the rigor of the results yielded by the current meta-analytic study.

We scanned the titles of documents using the following search string to identify data: (‘instructional leader*’ OR ‘instructional leadership’ OR ‘instructional manage*’ AND ‘self-efficacy’ OR ‘self-efficacy’ OR ‘teacher* self-efficacy’ OR ‘teacher* self efficacy’).

Our inclusion/exclusion criteria for the selection of studies were as follows:

*Inclusion Criteria:* (1) studies involving eligible statistical data for meta-analysis, and (2) studies measuring the relationship between PIL-TSEF or PIL-CEF; *Exclusion Criteria:* (1) studies with missing correlational values; (2) conceptual/review papers; and (3) studies in a language other than English or Turkish.

**Figure 2:**
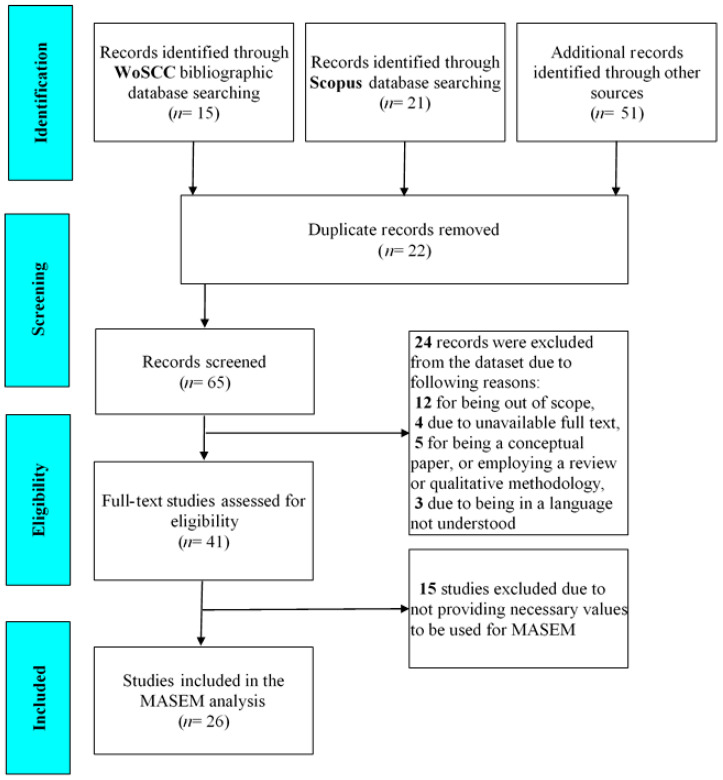
PRISMA flow diagram.

The initial search yielded a total of 87 studies: 15 from WoS, 21 from Scopus, and 51 from other sources (e.g., ERIC, Google Scholar, etc.). After identifying the duplicates, a total of 65 documents were left to screen for eligibility. Among these 65 documents, 24 documents were excluded from the dataset due to the following reasons: 12 studies were out of scope (e.g., principal efficacy for IL, shared instructional leadership, etc.), 4 studies did not have a full text available, 5 were conceptual papers or involved a review or qualitative methodology, and 3 studies were in a language not understood by the researchers (i.e., 2 Chinese and 1 Arabic). At the end of this process, a total of 41 documents were left for detailed screening. Among these, 15 documents were not included in the final analysis due to not providing the necessary values to be used for MASEM (correlation values in particular). Consequently, values from a total of 26 documents (22 articles and 4 dissertations) were included in the MASEM analysis (Figure 2).

### 3.2. Coding Procedure

Before the analysis of the 26 documents selected for this study, these documents were subjected to a coding procedure to identify the details regarding the purpose, methodology, population, measurement tools, context, and type of documents. These results are presented in Table 1.

Table 1 demonstrates that the studies mostly focused on analyzing the relationship between PIL and TSEF, CEF, and other teacher- or student-level variables such as teacher commitment or student achievement. The population samples in these studies ranged between 36 and 6019, and they included a total of 19,584 participants. The studies were correlational and consisted of 4 dissertations and 22 articles.

### 3.3. Data Analysis

Before testing the mediation model of the perceived CEF between PIL and TSEF, the overall effect sizes between PIL, CEF, and TSEF were calculated. The overall effect sizes were then transformed into Pearson’s *r* following Fisher’s Z transformation. Since Pearson’s *r* has a value in the range of ±1, it exhibits a non-normal distribution at values greater than 0.25 as an absolute value [90]. The overall effect sizes were calculated according to the random-effect model [91] since the studies included in this review were collected from the literature. However, the overall effect sizes that were calculated according to the common (fixed)-effect model for sensitivity analysis are also reported. The analysis yielded close values for overall effect sizes when calculated according to the common (fixed)- and random-effect models, providing evidence of their robustness and reliability.

The effect sizes were interpreted using the effect size scale recommended by Lovakov and Agadullina [92] for social psychology. Accordingly, the effect sizes of 0.12, 0.24, and 0.41 were interpreted as ‘small’, ‘medium’, and ‘large’, respectively. In addition, the prediction interval [91] was calculated to determine how true effects were distributed around the overall effect size. Additionally, a heterogeneity test was conducted to determine the presence and magnitude of variance between studies. The fact that the result of this test is significant (*p* < 0.05) indicates the presence of heterogeneity. Its size was determined by using the *I^2^* index. According to Higgins et al. [93], the *I*^2^ index of up to 25% indicates ‘low’ heterogeneity, while a value of up to 50% indicates ‘medium’ heterogeneity, and a value of up to 75% indicates ‘high’ heterogeneity.

To determine the source of heterogeneity, categorical moderator analysis was conducted according to the type of scale used to measure PIL and the publication type. On the other hand, meta-regression was conducted to determine whether the percentage of female participants had a significant relationship with the effect sizes. Sensitivity analysis was performed to evaluate whether there was any publication bias. For this purpose, contour funnel plots are presented. In these plots, studies with a 99% confidence interval are shown in ‘dark gray’, studies with a 95% confidence interval are shown in ‘light gray’, and studies with a 90% confidence interval are shown in ‘white’. The significance of the asymmetry in the funnel plot was tested using Egger’s regression test [94] and rank correlation test [95], while a trim-and-fill analysis [96] was performed to calculate the unbiased overall effect size. These analyses were carried out using the meta package [97] on the R platform [98].

To test the proposed mediation model, the two-stage structural equation modeling approach (TSSEM) developed by Cheung and Chan [64] and Cheung [99] was used. In the first stage of this analysis, an average correlation matrix was created according to the random-effect model. In the second stage, a structural equation model was established using this matrix. The *average correlation matrix* and *variance* were calculated according to the *maximum likelihood estimation* method, and the estimates of the structural equation model were calculated according to the *weighted least square estimation* method. The *one-stage meta-analytic structural equation modeling* (OSMASEM) method recommended by Jak and Cheung [100] was used to determine whether study characteristics (type of leadership scale, percentage of female participants, and publication type) were moderators of the model’s regression coefficients. The metaSEM [101] package was used on the R platform to test the mediation model. OSMASEM was carried out using the codes provided in the study conducted by Schutte et al. [102].

## 4. Results

### 4.1. Overall Effect Size between PIL and TSEF

**Figure 3:**
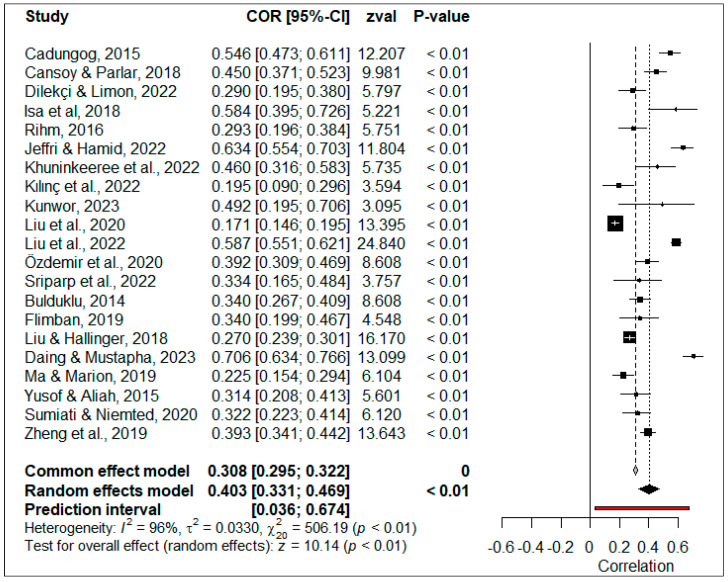
Forest plot of correlation between PIL and TSEF [6,10,18,20,29,44,55,68,71,72,73,74,75,77,79,80,81,82,84,86,87].

The correlation coefficients obtained from a total of 17,170 participants from 21 studies were used to calculate the general effect size of the relationship between PIL and TSEF. The forest plot of the effect sizes is illustrated in Figure 3.

The forest plot presented in Figure 3 demonstrates that all the correlation coefficients included in the meta-analysis have a significant (*p* < 0.05) effect size. When studies were combined according to the random-effect model, the overall effect size was calculated as 0.403, 95% [0.331, 0.469]. This overall effect size can be interpreted as ‘large’ according to the Lovakov and Agadullina scale [92]. The overall effect size was positive and significant (*p* < 0.05), indicating that the perceived TSEF increased in parallel to the perceived PIL. The estimation range showed that the true effect sizes were distributed around an overall effect size ranging between 0.036 and 0.674. The absence of a ‘zero’ effect size in this distribution showed that this relationship was positive and significant in 95% of all population samples.

The heterogeneity test result indicated that the heterogeneity of the studies included in the research was significant, with 20 degrees of freedom (*χ*^2^ = 506.19, *p* < 0.05), and the magnitude of heterogeneity was high (*I*^2^ = 96%) according to Higgins et al.’s scale [93]. The results of the categorical moderator analysis conducted to determine the source of heterogeneity according to the type of scale used to measure PIL and publication type are presented in Table 2.

The results presented in Table 2 indicate that the type of scale was a significant (*p* < 0.05) moderator, whereas the publication type was not (*p* > 0.05). Accordingly, the relationship between PIL and TSEF varied depending on the scale used to measure PIL. In particular, the measurements determined using the IL scale tended to yield a lower level of relationship than those obtained with other scales. However, the statistical power of the analysis was weak since only one measurement result was obtained from scales other than the PIMRS. The fact that the effect sizes do not differ significantly according to the publication type can be interpreted as the quality of the dissertations and articles included in the analysis being similar.

**Figure 4:**
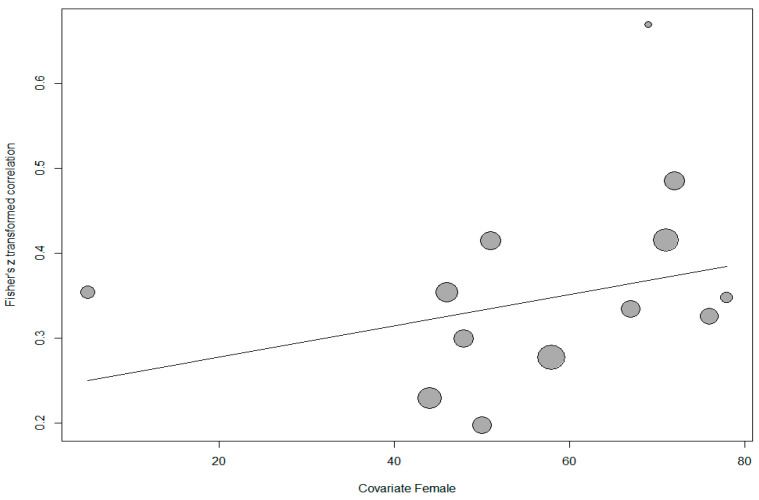
Scatter plot of correlation between PIL and TSEF.

In addition, meta-regression was conducted to determine whether the percentage of female participants had a relationship with the effect sizes. However, nine studies were excluded from the analysis because the percentage of female participants was not reported. The scatter plot showing the distribution of effect sizes according to the percentage of female participants is shown in Figure 4.

The results of the analysis performed to determine whether the distribution in the scatter plot was significant indicated that the model explaining the effect size distribution relative to the percentage of female participants was not significant (*Q*(1) = 1.524, *p* > 0.05). Based on this result, it could be inferred that the percentage of female participants was not a significant predictor of the relationship between PIL and TSEF (*p* > 0.05).

**Figure 5:**
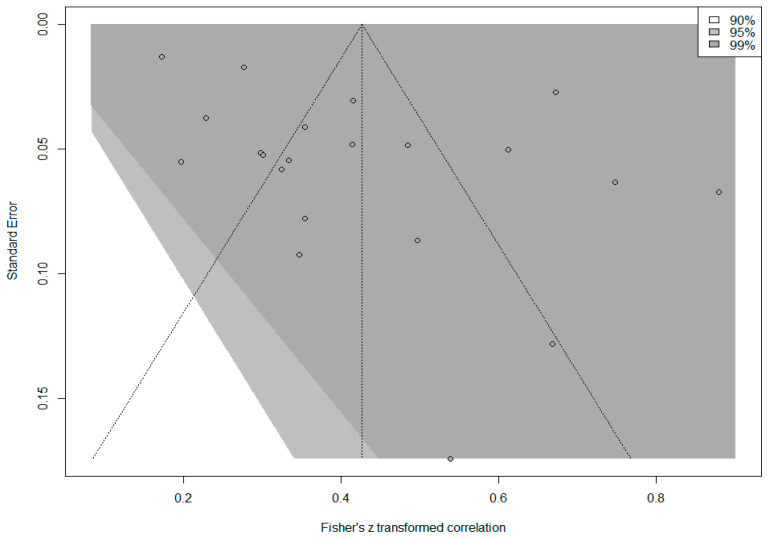
Funnel plot of the correlation between PIL and TSEF.

The contour funnel plot presented in Figure 5 was examined to determine whether the calculated overall effect size was the product of publication bias.

The funnel plot in Figure 5 shows that the effect sizes of all studies included in the meta-analysis are within the 99% confidence interval. The effect sizes have an asymmetric distribution around the mean and outside the boundaries of the funnel plot. *Egger’s regression test* and *rank correlation test* were performed to determine whether this asymmetry was significant. The results of Egger’s regression test (*p* = 0.058) and the rank correlation test (*p* = 0.205) showed that the asymmetry was not statistically significant (*p* > 0.05). Since complete symmetry was achieved in the funnel plot, a *trim-and-fill analysis* was performed to determine the unbiased effect size. Notably, 11 imputed studies were included in this analysis to obtain an unbiased effect size, and the expected overall effect size was calculated as 0.242 95% [0.137, 0.342]. While the observed overall effect (*r* = 0.403) was ‘large’, the expected overall effect (*r* = 0.242) decreased to a ‘medium’ level, which indicates that there may be publication bias. Therefore, it is necessary to take caution in interpreting the overall effect size.

### 4.2. Overall Effect Size between PIL and CEF

**Figure 6:**
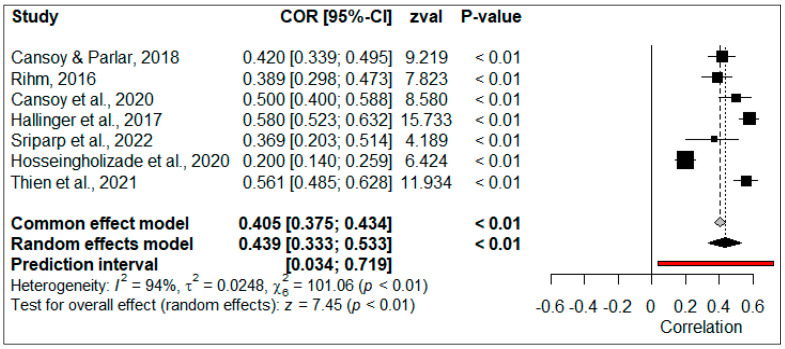
Forest plot of correlation between PIL and CEF [21,22,29,73,78,80,89].

The correlation coefficients obtained from seven studies (*N* = 3091) were used to calculate the overall effect size of the relationship between PIL and CEF. The forest plot of effect sizes is shown in Figure 6.

As seen in the forest plot, the effect sizes of all studies included in the meta-analysis are significant (*p* < 0.05). When effect sizes were combined according to the random-effect model, the overall size was calculated as 0.439, 95% [0.333, 0.533]. This effect size was positive, significant (*p* < 0.05), and ‘large’, indicating that the level of perceived SEF increased in parallel to perceived PIL. The estimation range showed that the true effect sizes were distributed around an overall effect size between 0.034 and 0.719. The absence of a 0 (*zero*) effect size in this distribution indicated that this relationship was positive and significant in 95% of all populations.

The results of the heterogeneity test show that the heterogeneity of the studies included in the research was significant, with six degrees of freedom (*χ*^2^ = 101.06, *p* < 0.05), and the magnitude of heterogeneity was high (*I*^2^ = 94%). A categorical moderator analysis was conducted to determine the source of variance according to the type of scale used to measure PIL (PIMRS vs. ESL) and publication type (article vs. dissertation). The result of this analysis is presented in Table 3.

As presented in Table 3, the results of the categorical moderator analysis showed that neither the type of the scale (*Q*(1) = 32.640, *p* > 0.05) nor the publication type (*Q*(1) = 3.460, *p* > 0.05) was a significant moderator.

**Figure 7:**
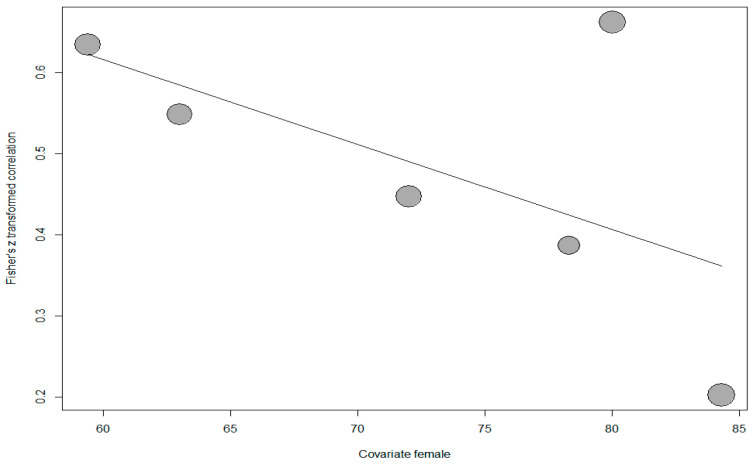
Scatter plot of the distribution of effect sizes according to the percentage of female participants.

On the other hand, a meta-regression analysis was conducted to determine whether the percentage of female participants had a relationship with the effect sizes. One study was excluded from the analysis because the percentage of female participants was not reported. The scatter plot showing the distribution of effect sizes according to the percentage of female participants is shown in Figure 7.

The analysis performed to determine whether the distribution in the scatter plot in Figure 7 was significant showed that the model explaining the distribution of effect sizes relative to the percentage of female participants was not significant (*Q*(1) = 2.034, *p* > 0.05). This result indicates that the percentage of female participants is not a significant predictor of the relationship between PIL and CEF.

**Figure 8:**
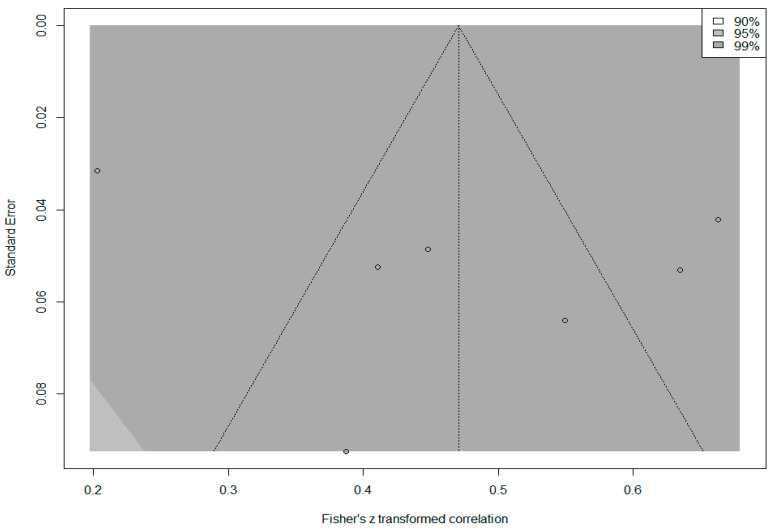
Contour funnel plot of the correlation between PIL and CEF.

In addition, the *contour funnel plot* in Figure 8 was examined to determine whether the calculated overall effect size was the product of publication bias.

The funnel plot in Figure 8 indicates that the effect sizes of all studies included in the meta-analysis are within the 99% confidence interval. The effect sizes showed an asymmetric distribution around the mean and outside the boundaries of the funnel plot. The fact that the results of Egger’s regression test (*p* = 0.295) and the rank correlation test (*p* = 0.881) were not statistically significant (*p* > 0.05) indicated that this symmetry was not significant. To obtain an unbiased effect size, a trim-and-fill analysis was conducted, and three imputed studies were included in the analysis. The expected overall effect size was calculated as 0.317, 95% [0.163, 0.457]. While the observed overall effect (*r* = 0.44) was at a ‘large’ level, the expected overall effect (*r* = 0.32) decreased to a ‘medium’ level, which indicates that there may be publication bias.

### 4.3. Overall Effect Size between CEF and TSEF

**Figure 9:**
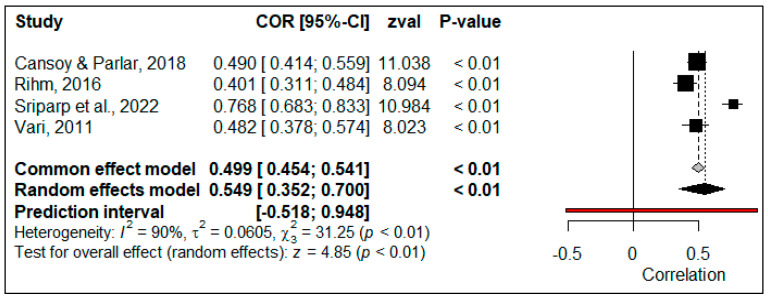
Forest plot of correlation between CEF and TSEF [29,73,80,83].

To calculate the overall effect size of the relationship between CEF and TSEF, correlation coefficients obtained from four studies (*N* = 1149) were used. The forest plot of the effect sizes is shown in Figure 9.

The forest plot in Figure 9 demonstrates that the effect sizes of all studies included in the meta-analysis are significant (*p* < 0.05). When the effect sizes were combined according to the random-effect model, the overall size was calculated as 0.549, 95% [0.352, 0.700]. This effect size was positive, significant (*p* < 0.05), and ‘large’, indicating that the level of perceived TSEF increased in parallel to perceived CEF. The estimation range shows that the true effect sizes are distributed around an overall effect size between -0.518 and 0.948. The fact that this distribution varied between negative and positive effect sizes indicated that it was not significant in all populations (*p* > 0.05). Therefore, the effect size of CEF did not have a significant relationship with TSEF in all populations.

The results of the heterogeneity test showed that the heterogeneity of the studies included in this research was significant, with three degrees of freedom (*χ*^2^ = 31.25, *p* < 0.05), and the magnitude of heterogeneity (*I*^2^ = 90%) was at a high level. To determine the source of variance, a categorical moderator analysis was conducted according to the type of scale used to measure PIL and the publication type (article vs. dissertation). The results of this analysis are presented in Table 4.

As presented in Table 4, the results of the categorical moderator analysis showed that neither the type of scale (*Q*(1) = 32.640, *p* > 0.05) nor the publication type (*Q*(1) = 3.460, *p* > 0.05) were significant moderators. However, meta-regression could not be performed because the number of included studies was very small.

**Figure 10:**
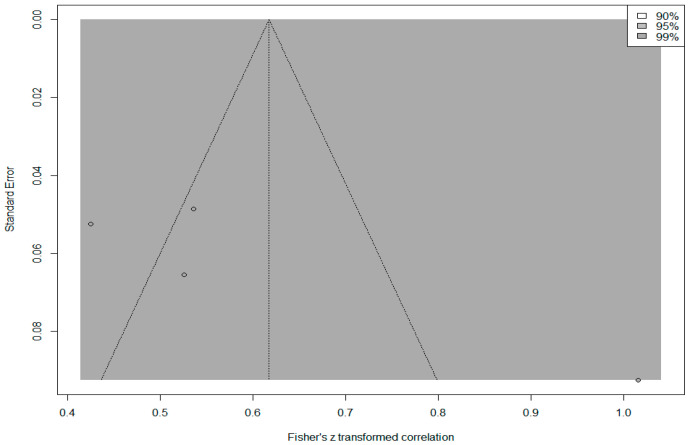
Contour funnel plot of the correlation between CEF and TEF.

The contour funnel plot presented in Figure 10 was examined to determine whether the calculated overall effect size was the product of publication bias.

The funnel plot in Figure 10 shows that the effect sizes of all studies included in the meta-analysis are within the 99% confidence interval. The effect sizes have an asymmetric distribution within and outside the boundaries of the funnel plot. The results of Egger’s regression test (*p* = 0.146) and the rank correlation test (*p* = 0.497) indicated that this asymmetry was not statistically significant (*p* > 0.05). Based on the results of the trim-and-fill analysis, the expected overall effect size was calculated as 0.549, 95% [0.352, 0.700]. Since the expected effect size is equal to the observed effect size, it can be concluded that there is no publication bias.

### 4.4. The Mediating Effect of CEF in the Association between PIL and TSEF

**Figure 11:**
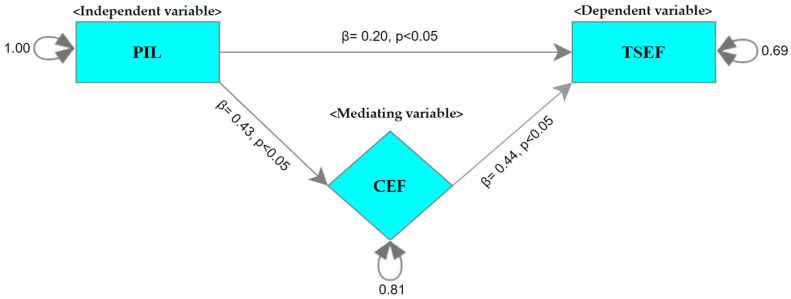
MASEM results for the proposed model. (Note. PIL = principal instructional leadership; TSEF = teacher self-efficacy; CEF = collective efficacy).

To test the mediating role of CEF in the association between PIL and TSEF, correlation matrices were created using the correlation coefficients obtained from the studies included in the present study. The results of the analysis performed to determine the heterogeneity of the correlation matrices showed that these matrices were heterogeneous (χ(29) = 634.99, *p* < 0.001). Therefore, in the first stage of MASEM, correlation matrices were combined according to the random-effect model. In the second stage of MASEM, the average correlation matrix calculated in the first stage was used to test the fit of the mediation model. The path graph of the mediation model is shown in Figure 11.

Since the mediation model was saturated with 0 (zero) degrees of freedom, no fit index was calculated. The path coefficient between PIL and CEF was calculated as 0.433, 95% [0.353, 0.521]; the path coefficient between PIL and TSEF was calculated as 0.196, 95% [0.166, 0.221]; and the path coefficient between CEF and TSEF was calculated as 0.444, 95% [0.363, 0.544]. As shown in the path graph in Figure 11, PIL alone explained 19% of the variance in CEF. The direct and indirect effects of PIL on CEF explained 31% of the variance in TSEF. While the direct effect of PIL on TSEF was calculated as 0.196, 95% [0.166, 0.221], its indirect effect was determined as 0.192, 95% [0.166, 0.221]. The indirect effect was significant at the 95% confidence interval (*p* < 0.05), revealing that CEF was a partial mediator in the association between PIL and TSEF. The analysis of variance (ANOVA) performed to determine whether the direct and indirect effects of PIL differed significantly showed that the difference was not statistically significant (χ(1) = 0.006, *p* > 0.05). These results indicate that the direct and indirect effects of PIL have a similar magnitude.

A moderator analysis was conducted to determine whether the characteristics of the primary studies included in this review predicted the path coefficients. As categorical moderators, the type of scales used to measure PIL and the publication type (article and dissertation) were used. The percentage of female participants in the primary studies was used as a continuous variable. The scales used to measure PIL were tested for their predictive effect on the path coefficients between PIL and CEF and between PIL and TSEF. The results of the test showed that the path coefficients of the scales, both between PIL and CEF (χ(5) = 0.643, *p* = 0.642) and between PIL and TSEF (χ(5) = 3.440, *p* = 0.632), were not significant predictors (*p* > 0.05). One reason why the type of scales did not explain the variance may be that the majority of the scales used to measure PIL were the PIMRS and one from each of the other five scale types. Similarly, according to the results of the moderator analysis conducted to determine whether the percentage of female participants and the publication type were significant moderators in predicting the path coefficients, both the percentage of female participants (χ(5) = 6.865, *p* = 0.08) and the type of publication (χ(5) = 3.043) *p* = 0.385) were not significant moderators (*p* > 0.05).

## 5. Discussion

This study aimed to investigate the mediating role of CEF in the association between PIL and TSEF. For this purpose, correlation coefficients were collected from 26 studies (*N* = 19,584) examining the relationships between PIL, CEF, and TSEF from the literature. Before testing the mediation model, the overall effect sizes of the relationships between PIL-TSEF, PIL-CEF, and CEF-TSEF were calculated. The mediation model was later tested with a two-stage structural equation modeling approach (TSSEM). In the first stage of this approach, the average correlation matrix was created according to the random-effect model, and in the second stage, the regression coefficients for the model were calculated.

The general effect size of the association between PIL and TSEF was calculated by combining the correlation coefficients obtained from a total of 17,170 participants from 21 studies according to the random-effect model, and the overall effect size was calculated as *r* = 0.40. This result showed that the perceived TSEF increased in parallel to the perceived PIL at a ‘large’ level. This finding indicates that school principals influence the perceptions of teachers regarding their ability to attain educational goals via instructional leadership practice. By establishing goals and a vision around clear expectations of high-quality teaching and learning, providing direction and supervision as well as being available to support teachers any time by any means, monitoring student progress and classroom instruction, and creating a positive culture of learning at school, principals can promote teachers’ beliefs in their ability to make a meaningful change in students’ learning [17,22,44,53,54,77,103,104]. For instance, Duyar et al. [29] found that the supervision provided by principals is very significant in supporting TSEF. From a reverse perspective, Skaalvik and Skaalvik [105] found that the absence or insufficiency of supervisory support from the principal affected TSEF negatively.

In his conceptualization of self-efficacy, Rotter [106] explained that efficacy beliefs are built over perceptions of internal and external control. PIL supports teachers’ perceived internal control by providing teachers with the materials and conditions to employ better instruction and creating opportunities for professional growth, all of which are likely to enhance teachers’ perceptions of internal control over their teaching. Similarly, principals as instructional leaders support teachers when they face challenging situations, set clear goals and standards for achievement, and collaborate with teachers to improve their instruction and monitor and acknowledge achievements, which in turn support the perceived external control of teachers. For instance, Anderson [107] stated that when principals work collaboratively with teachers and convey a message of confidence in their teaching capabilities, they significantly support their self-efficacy. Liu et al. [10] on the other hand, underlined that PIL can enhance TSEF by supporting teachers’ leadership in instruction, which might enhance their perceived internal control of instruction. Similarly, Herewati et al. [108] suggested that PIL significantly supports teachers’ professional competence, and this helps enhance TSEF.

The general effect size of the association between PIL and CEF was calculated by combining the correlation coefficients obtained from a total of 3091 participants from seven studies according to the random-effect model, and the overall effect size was calculated as *r* = 0.44. This result indicated that as the perceived PIL increased, the perceived CEF also increased at a ‘large’ level. Research shows that PIL enhances CEF by establishing a culture of cooperation and sharing among teachers [28,48,109]. The synergy resulting from higher levels of cooperation and collaboration facilitates perceived CEF [57,110] as teachers’ joint efforts and collaborative experiences ‘demonstrate what “we, as teachers of this school” can accomplish together’ [46] (p. 1404). Research also indicates that PIL increases teachers’ participation in peer observations, principal coaching, and teacher mentoring [111], which is likely to support CEF by increasing teachers’ perception that they are working in a supportive environment [60,111,112,113] and by enhancing their ability to take the necessary actions to provide better learning outcomes [27].

The general effect size of the association between CEF and TSEF was calculated by combining the correlation coefficients obtained from a total of 1149 participants from four studies according to the random-effect model, and the overall effect size was calculated as *r* = 0.55. This result showed that as the perceived CEF increased, the perceived TSEF also increased at a ‘large’ level. According to Bandura’s concept [26], enactive experiences, social persuasion, vicarious learning, and affective states are the four main sources of efficacy. Accordingly, as teachers’ perceptions of individual and collective mastery increase, their efficacy beliefs are strengthened [114]. In light of social cognitive theory, Bandura [26] also proposed that a reciprocal causality exists between what teachers believe they can do collectively and the individual teacher’s cognitive, social, and behavioral competencies. Together, they help increase their motivation and ability to improve their teaching. In line with the findings of the current study, the results of other studies also support this postulation [115,116]. Similarly, as increased collaboration and CEF increase the level of student achievement, it is very likely to support teachers’ individual beliefs about how they can make a positive change in students’ learning [117].

The results of meta-analytic structural equation modeling (MASEM) provided evidence indicating that CEF was a partial mediator in the association between PIL and TSEF. In other words, CEF partially explained the relationship between PIL and TSEF. Accordingly, the direct effect of PIL on TSEF was *β* = 0.20, and its indirect effect was *β* = 0.19. The results of the variance analysis showed that the difference between these effects was not significant (*p* > 0.05), indicating that the direct and indirect effects had a similar magnitude.

The social cognitive theory forming the backbone of both TSEF and CEF postulates that human agency is shaped by the juxtaposition of personal, behavioral, and environmental factors [118]. From this perspective, our results indicate that the social support provided by PIL enhances CEF, and these two variables (PIL and CEF) together enhance TSEF by providing a supportive work environment [46,116,119].

Studies show that as the level of principal’s guidance and supervision for instruction increases, both the collective efficacy and individual efficacy of teachers increase. In addition, CEF becomes a significant mediating variable between PIL and TSEF [21,89]. As eloquently articulated by Viel-Ruma et al. [120], ‘When teachers as a group… believe that the staff as a whole can be successful, they will be more likely to persist in their efforts to achieve such success’ (p. 227). Similarly, PIL is considered to improve teachers’ professional learning and competence by providing teachers with a social capital, namely their peers with whom they can share ideas and work together. As postulated by social capital theory, PIL helps to develop CEF by increasing networks and collaboration among teachers, which in turn supports teachers’ individual skills to become better teachers [108]. These arguments also support our finding that while PIL alone explained 19% of the variance in CEF, PIL and CEF together explained 31% of the variance in TSEF. The regression coefficients of the model showed that PIL affected TSEF at a ‘small’ level (β = 0.20) and CEF at a ‘large’ level (β = 0.43).

Moderator analyses were conducted to determine whether the relationships and regression coefficients between PIL, CEF, and TSEF were predicted by the characteristics of the studies (the type of PIL scale, publication type, and the percentage of female participants). The results indicated that only the relationship between PIL and TSEF differed significantly depending on the type of scale used and showed that the IL scale measured the relationship at a lower level than other scales. One possible reason for this difference may be the fact that the PIMRS evaluates teachers’ perceptions of PIL on the basis of the frequency of several leadership acts (from almost always to never) combined under three main components, i.e., defining school mission, managing curriculum, and promoting school climate [37]. Other scales, on the other hand, evaluate the extent to which teachers agree that their principal employs particular leadership tasks (e.g., [76,88]). The scale established by Lineburg [69], on the other hand, provides more detailed items on principals’ frequency of promoting professional development; providing resources, incentives, and support; supervising instruction; and issuing directives. The scale is more controlled in terms of the received responses as the number of weeks/months is specified corresponding to each category of response from always to never. The different levels of focus among scales as well as the differences in their wording of items could have affected the measure of teachers’ PIL practice in their schools.

However, the publication type was not found to be a significant moderator for effect sizes and regression coefficients. Publication type can be an indicator of the quality of studies [102]. Accordingly, it can be stated that the quality of the dissertations and articles included in the current study was similar. In addition, the percentage of female participants did not significantly predict the effect sizes or regression coefficients (*p* > 0.05). In fact, in leadership studies, whether female and male leaders perform leadership differently has been a topic of interest [121]. In the context of PIL, research even suggested that female leaders could practice PIL more often than males due to being more relationship-oriented and collaboration-centered [15]. In their review limited to studies using the PIMRS, Hallinger et al. [122] found a small but statistically significant effect on PIL, with more active PIL from female principals (p. 23). However, none of these studies focused on the association between PIL and CEF/TSEF. Studies on teachers’ self-efficacy have also reported some conflicting results. For instance, Kılınç et al. [6] found that PIL supported the self-efficacy of female teachers slightly stronger than that of males. However, when they evaluated the gender effect according to subdimensions of TSEF, they found no significant difference. They also stated that male teachers perceived a slightly higher level of PIL than female teachers. Some other studies showed that female teachers had lower levels of self-efficacy than males [10,86] whereas some controlled for gender in their study while exploring the association between PIL and TSEF [83]. Another interesting finding from Rihm [73] showed that the context and location of schools influence the effect of gender on the perceived PIL and efficacy.

In addition to these results, previous studies also showed that some variables other than gender could be more influential on the efficacy beliefs of teachers, regardless of whether these teachers were male or female. One such variable may be the culture of the school or the society that demonstrates different gender attributes [25,123,124,125,126]. Similarly, despite some existing controversy, scholars have also underlined that TSEF is usually correlated with teaching experience, not only in terms of the length of the experience but also in terms of the quality of the teaching experience [116,127,128,129]. In addition, some studies indicated that lower or higher years of experience could negatively affect TSEF, and TSEF could be optimum during mid-career [130]. Some other studies even indicated that the gender mix in the classroom could influence the efficacy beliefs of female and male teachers differently [131]. Accordingly, as classrooms become more heterogeneous regarding gender, the difference between male and female teachers’ self-efficacy might decrease or become insignificant. These results suggest that the influence of gender on TSEF should be investigated by including a larger variety of demographic variables to be able to observe its clear and direct effect on TSEF.

These controversial results do not provide a significant ground for the findings of the present study and indicate that more work is warranted on the moderating role of gender in the association between PIL, CEF, and TSEF. Another point to iterate is the fact that the studies included in the present analysis mostly involved female participants, and several of the studies did not give detailed information about the gender of their sample, which might have affected the results of the analysis.

### Limitations and Implications for Future Research

The overall effect size of the association between PIL and TSEF showed significant differences under the common (fixed)- and random-effect models. This may be due to the high degree of variability in the studies selected for this study. In addition, extreme values across studies may also contribute to this difference. This may ultimately affect the robustness of the overall effect size. Another concern that threatens the reliability of this effect size is the publication bias effect. Although the asymmetric distribution in the funnel plot was not significant, the results of the trim-and-fill analysis showed that the expected effect size also differed significantly from the observed effect size. Therefore, it is necessary to take caution in interpreting the results based on this overall effect size. The effect sizes related to PIL and TSEF differed significantly depending on the type of scale used. However, since only one measurement result was obtained from scales other than the PIMRS, the statistical power of the analysis remains weak.

A small number of studies were included in this review to calculate the overall effect sizes between TSEF, CEF, and PIL. To calculate a more robust overall effect size, more primary studies investigating the relationship between these variables are needed. In particular, the range of estimates for the effect sizes between CEF and TSEF vary between negative and positive, indicating that perceived CEF might not affect perceived TSEF in all populations; in some populations, there may even be an inverse relationship. In addition, although the heterogeneity between these effect sizes was high, the identified moderators could not be explained by the selected variables. For this reason, conducting research synthesis studies [132] by including both qualitative and quantitative studies in future meta-analytic studies can make significant contributions to the relevant field of research. Similarly, our data search did not yield a significant amount of research from Western contexts, and data were particularly lacking from European perspectives. Although we put special effort into reaching a high variety of contexts, our data search did not yield research from these contexts. This has implications both for the future investigation of these variables in Western contexts and also for the limited generalizability of our results for these Western contexts.

The path coefficients calculated in the tested mediation model revealed that PIL could affect TSEF at a ‘small’ level and CEF at a ‘large’ level. However, since these coefficients are relationship coefficients, they do not allow for a direct inference regarding causality, because this theoretical relationship may also be bidirectional. In other words, higher CEF may have led to higher perceived PIL. Therefore, in future research, longitudinal and experimental studies should be conducted to better understand the causal relationships between these variables.

The results of the current study also have some implications for policy and practice as well as for future investigations involving these aspects. Our results provide evidence from a more global perspective indicating that PIL is significant in supporting CEF and TSEF, which are crucial precursors in achieving educational goals. Therefore, educational policies should continue to emphasize instructional leadership responsibilities as important job qualifications of principals while at the same time making these responsibilities a central part of principal training and evaluation initiatives. Similarly, not only school principals but also all other leaders involved in the hierarchy of educational systems should strive to increase CEF and TSEF, as in Jerald’s words [133], ‘what is most promising about this line of research is that (the) efficacy perceptions are not set in stone’ but could be improved toward creating better educational outcomes.

As for the future investigations of PIL, CEF, and TSEF, our search for data showed that the number of studies attempting to understand their interrelationships as well as the effect of these relationships on other teacher- or school-level variables such as teacher commitment, job satisfaction, professional learning, and school climate [9,60,134,135] is quite limited in the literature. Although there are some lines of evidence indicating that TSEF or CEF has a significant influence on student outcomes as well as teacher-related variables, studies evaluating their combined effects and reciprocal relationships are warranted to support this field of research.

## 6. Conclusions

Sustaining the improvement and effectiveness of schools in the complex and fast-changing context of the twenty-first century is one of the central concerns in educational studies, and leadership has become a focus of research interest as a means of supporting the quality of student outcomes. Among the leadership models, PIL was found to be the most significant leadership model to improve a range of student outcomes since PIL improves the conditions for quality instruction and learning, which in turn is likely to support students’ achievement [35,136]. As leadership is ultimately practiced by influencing the followers [137], principals’ instructional leadership can positively influence the behaviors and perceptions of teachers by establishing a more supportive and collaborative culture in school. This makes PIL a significant variable in supporting CEF and TSEF. The relationship between these variables, on the other hand, matters since the literature presents a well-established relationship between these variables and student growth as the ultimate goal of education [57].

## Figures and Tables

**Table 1:** Details of the studies included in the analysis.

					Gender				
No.	Author(s), Year	Research Focus	Method	Sample Size	Female	Male	Scales for PIL	Country	Type
1	Cadungog, 2015 [68]	The mediating role of professional development in the relationship between PIL and TSEF	Correlational	400	n.a.	n.a.	Instructional Leadership Questionnaire—ILQ (Lineburg, 2010 [69])	Philippines	Article
2	Cansoy and Parlar, 2018 [29]	The relationship between PIL, CEF, and TSEF	Correlational	427	72%	28%	Effective School Leadership Scale—ESLS (Ata, 2015 [70])	Türkiye	Article
3	Dilekçi and Limon, 2022 [71]	The mediating role of TSEF in the relationship between PIL and teachers’ positive instructional emotions (enjoyment, pride, hope)	Correlational	380	47.60%	52.40%	Principal Instructional Management Rating Scale—PIMRS (Hallinger and Murphy, 1985 [37])	Türkiye	Article
4	Isa et al., 2018 [72]	The relationship between PIL and TSEF	Correlational	64	68.75%	31.25	PIMRS	Malaysia	Article
5	Rihm, 2016 [73]	The relationship between PIL and academic heterogeneity with TSEF	Correlational	366	n.a.	n.a.	PIMRS	Chile	Dissert.
6	Jeffri and Hamid, 2022 [74]	The relationship between PIL and TSEF	Correlational	252	n.a.	n.a.	PIMRS	Malaysia	Article
7	Khun-inkeeree et al., 2022 [75]	The relationship between PIL and TSEF	Correlational	136	n.a.	n.a.	PIMRS	Malaysia	Article
8	Cansoy et al., 2020 [21]	The mediating role of CEF in the relationship between PIL and teacher commitment	Correlational	247	63%	37%	IL Scale (Alig-Mielcarek, 2013 [76])	Türkiye	Article
9	Kılınç et al., 2022 [6]	The relationship between PIL and TSEF	Correlational	334	49.40%	50.60%	PIMRS	Türkiye	Article
10	Kunwor, 2023 [77]	The effects of PIL on TSEF	Correlational	36	n.a.	n.a.	PIMRS	Nepali	Article
11	Hallinger et al., 2017 [78]	The relationship between principal self-efficacy, PIL, TSEF, and teacher commitment	Correlational	567	80%	20%	PIMRS	Iran	Article
12	Liu et al., 2020 [18]	The effect of PIL/DL on TSEF and job satisfaction; the role of supportive school culture and teacher collaboration	Correlational	6019	n.a.	n.a.	PIMRS	International	Article
13	Liu et al., 2022 [10]	The relationship between instructional leadership and TSEF/student achievement	Correlational	1365	n.a.	n.a.	PIMRS	China	Article
14	Özdemir et al., 2020 [79]	The effects of PIL on TSEF	Correlational	435	51%	49%	PIMRS	Türkiye	Article
15	Sriparp et al., 2022 [80]	The relationship between PIL and TSEF, and the moderation of teacher role	Correlational	120	78.3%	21.70%	PIMRS	Thailand	Article
16	Bulduklu, 2014 [81]	The relationship between PIL, TSEF, and student achievement	Correlational	594	46%	54%	PIMRS	Türkiye	Dissert.
17	Flimban, 2019 [82]	The effect of PIL on TSEF in elementary schools	Correlational	168	5.35%	94,64%	PIMRS	The USA	Dissert.
18	Vari, 2011 [83]	The relationship between PIL and CEF	Correlational	236	83.75%	15.41%	PIMRS	The USA	Dissert.
19	Liu and Hallinger, 2018 [20]	TSEF as a mediator of PIL and teacher professional learning	Correlational	3414	57.5%	42.50%	PIMRS	China	Article
20	Daing and Mustapha, 2023 [44]	The relationship between PIL, TSEF, and teacher performance in senior high schools	Correlational	225	n.a.	n.a.	PIMRS	Philippines	Article
21	Ma and Marion, 2019 [84]	The effect of PIL on TSEF, and the mediating role of faculty trust	Correlational	714	44%	56%	PIMRS	China	Article
22	Hosseingholizade et al., 2020 [85]	The relationship between PIL, CEF, teacher commitment, and teacher professional learning in primary schools	Correlational	1007	84.30%	15.70%	PIMRS	Iran	Article
23	Yusof and Aliah, 2015 [86]	The relationship between perceived PIL, and TSEF	Correlational	300	76.43%	23.57%	PIMRS	Malaysia	Article
24	Sumiati and Niemted, 2020 [55]	The relationship between PIL and TSEF in private elementary schools	Correlational	339	67.30%	32.70%	PIMRS	Indonesia	Article
25	Zheng et al., 2019 [87]	The relationships between PIL, TSEF, and the professional learning community	Correlational	1082	71.1%	28.9%	ILS (Louis et al., 2010 [88])	China	Article
26	Thien et al., 2021 [89]	The relationship between PIL, teacher commitment, and the mediating role of CEF	Correlational	357	59.40%	40.60%	PIMRS	China	Article

**Table 2:** The results of categorical moderator analysis.

Name of the Moderator	k	Effect Size	95% CI.		Heterogeneity		
			Lower Limit	Upper Limit	*Qb*	*df*	*p*
Scale type	21	0.403	0.331	0.469	32.640	4	0.0001
ILQ	1	0.546	0.473	0.611			
ESLS	1	0.450	0.371	0.523			
PIMRS	17	0.403	0.319	0.481			
IL	1	0.195	0.090	0.296			
ILS	1	0.393	0.341	0.442			
Publication Type	21	0.403	0.331	0.469	3.460	1	0.063
Article	18	0.416	0.335	0.492			
Dissertation	3	0.325	0.272	0.376			

**Table 3:** The results of categorical moderator analysis.

Name of the Moderator	*k*	Effect Size	95% CI.		Heterogeneity		
			Lower Limit	Upper Limit	*Qb*	*df*	*p*
Scale type	7	0.439	0.333	0.533	32.640	1	0.763
ESLS	1	0.420	0.339	0.495			
PIMRS	6	0.442	0.317	0.551			
Publication Type	7	0.439	0.333	0.533	3.460	1	0.438
Article	6	0.447	0.325	0.554			
Dissertation	1	0.389	0.298	0.473			

**Table 4:** The results of categorical moderator analysis.

Name of the Moderator	*k*	Effect Size	95% CI.		Heterogeneity		
			Lower Limit	Upper Limit	*Qb*	*df*	*p*
Scale type	4	0.594	0.352	0.700	0.390	2	0.821
ESLS	1	0.490	0.414	0.559			
OTHER	1	0.482	0.338	0.574			
PIMRS	2	0.614	0.136	0.860			
Publication Type	4	0.594	0.352	0.700	3.460	1	0.438
Article	2	0.647	0.291	0.845			
Dissertation	2	0.436	0.354	0.511			

## Data Availability

The original contributions presented in the study are included in the article, further inquiries can be directed to the corresponding author/s.
